# A RBM47 and IGF2BP1 mediated circular FNDC3B-FNDC3B mRNA imbalance is involved in the malignant processes of osteosarcoma

**DOI:** 10.1186/s12935-023-03175-3

**Published:** 2023-12-21

**Authors:** Congya Li, Linchao Ding, Xuyao Wang, Peng Shu, Xuchao Shi, Zhijian Zheng, Jian Liu, Junlan Zhu

**Affiliations:** 1 Precision Medicine Laboratory, Beilun People’s Hospital, Beilun Branch of the First Affiliated Hospital, School of Medicine, Zhejiang University, No.1288 of Lushan Road, Beilun District, Ningbo, 315800 Zhejiang China; 2 Health Science Center, Ningbo University, Ningbo, 315021 Zhejiang China; 3 Department of Scientific Research, Affiliated Jinhua Hospital, Zhejiang University School of Medicine, 365 Renmin East Road, Jinhua, 321000 Zhejiang China; 4 Department of Orthopaedics Surgery, Beilun People’s Hospital, Ningbo, Zhejiang China; 5 Department of Medical Oncology, Affiliated Jinhua Hospital, Zhejiang University School of Medicine, Jinhua, 321000 China

**Keywords:** Circular RNA, Osteosarcoma, FNDC3B, RBM47, IGF2BP1

## Abstract

**Background:**

Circular RNAs (circRNAs) are a class of noncoding RNAs that are involved in the progression of many human cancers. The precise gene locus and the roles of circular RNA from Fibronectin type III domain containing 3B (FNDC3B) in OS and its mechanisms of action have not been fully explored.

**Materials and methods:**

qRT-qPCR assay was used to determine gene expressions. CCK8 Assay, EdU assay, wound-healing assay, transwell invasion assay and in vivo xenograft assay were used to perform functional investigations. RNA-FISH, immunofluorescence, RIP assay, RNA stability analysis were applied in mechanistic studies.

**Results:**

We found that circFNDC3B downregulated and FNDC3B mRNA upregulated in OS, and might be potential biomarkers for indicating disease progression and prognosis of OS patients. CircFNDC3B acted as a tumor suppressor gene to restrain OS progression and FNDC3B functioned as an oncogene to promote OS progression in vitro and in vivo. RNA binding protein RNA binding motif protein 47 (RBM47) could bind to the flanking introns of circFNDC3B to facilitate the generation of circFNDC3B, resulting in the reduction of FNDC3B mRNA and the circFNDC3B-FNDC3B mRNA imbalance. CircFNDC3B also inhibited FNDC3B mRNA expression by reducing its stability via competitively binding to Insulin-like growth-factor-2 mRNA binding protein (IGF2BP1).

**Conclusion:**

This study demonstrated that RBM47 and IGF2BP1 mediated circular FNDC3B/FNDC3B mRNA imbalance was involved in the malignant processes of OS.

**Supplementary Information:**

The online version contains supplementary material available at 10.1186/s12935-023-03175-3.

## Introduction

Osteosarcoma (OS) is a prevalent and fatal primary bone malignancy that preferentially affects children and adolescents [1]. Although advanced therapy such as surgical resection combined with chemotherapy and/or radiotherapy has been applied, the prognosis of OS remained poor due to its high malignancy [2, 3]. The molecular mechanisms underlying the pathogenesis and development of OS are terribly complicated and largely unclear [4]. So it is necessary to illustrate the mechanical aspects of the OS to provide a novel approach to defeat OS.

Circular RNAs (circRNAs) are a class of noncoding RNAs with a closed-loop stable structure [5]. CircRNAs may exert multiple regulatory roles via a series of machinery, including transcriptional regulation, miRNAs or RNA-binding proteins (RBPs) interaction, or even serving as templates for translation [6]. An increasing number of studies have uncovered that circRNAs are involved in the pathogenesis and development of many human diseases, especially malignant tumors [7]. However, compared with other cancers, the research of circRNAs in osteosarcoma is relatively less, and their roles in osteosarcoma remain largely unknown.

RBPs are a class of proteins that are able to interact with multifarious RNAs by specific RNA-binding domains [8, 9]. These molecules always modulate the functions of RNAs by various patterns such as alternative splicing, altering stability and changing intracellular localization [8, 9]. The crosstalk between RBPs and circRNAs receives more and more attention recently. For example, RBP Quaking (QKI) may interact with the QKI response elements in the flanking introns of some circRNAs and thereby promoting the biogenesis of these circRNAs [10, 11]; circDLC1 functions as a tumor suppressor via binding to RNA-binding protein HuR, blocking the interaction between HuR and MMP1 mRNAs to restrain MMP1 expression in a competitive manner [12].

A recent study identified many circRNAs dysregulated in OS cell lines (U2OS, U2OS/MTX300, HOS, MG63, 143B, ZOS, and ZOSM) compared with human osteoblast hFOB1.19 cell line by analyzing GEO datasets [13]. Herein, we noticed that hsa_circ_0067971 was significantly downregulated in OS cell lines. Hsa_circ_0067971 originated from the Fibronectin type III domain containing 3B (FNDC3B) gene locus and was generated by the back-splicing of exon 2, so we termed it as circFNDC3B. Indeed, another circular RNA derived from exon 5 and exon 6 of FNDC3B gene, hsa_circ_0006156, has been verified to be a tumor suppressor in malignancies such as colorectal cancer [14, 15] and bladder cancer [16]. Nevertheless, the roles of circFNDC3B (hsa_circ_0067971) have not been reported in any cancers including OS. Moreover, there are also no studies on FNDC3B gene in OS at present. Therefore, we investigated circFNDC3B and FNDC3B in OS.

## Materials and methods

### Tissue samples

Fifty paired OS tissues and adjacent tissues were collected simultaneously from patients who underwent complete resection in Affiliated Jinhua Hospital, Zhejiang University School of Medicine. The clinical characteristics of OS patients were listed in Additional file 1: Table S1. The histological diagnosis was confirmed by two independent pathologists according to the criteria defined by the World Health Organization. All subjects gave their informed consent for inclusion before they participated in the study. The study was conducted in accordance with the Declaration of Helsinki, and the protocol was approved by the Ethics Committee of Affiliated Jinhua Hospital, Zhejiang University School of Medicine (2023-199).

### Cell culture

Human OS cell lines (U2OS, Saos-2, HOS, MG63 and 143B) and human osteoblast hFOB1.19 cell line were obtained from the American Type Culture Collection (ATCC, Manassas, VA, USA). OS cells were maintained in RPMI-1640 medium (HyClone, South Logan, UT, USA) supplemented with 1% penicillin, streptomycin and 10% fetal bovine serum (Invitrogen, Carlsbad, CA, USA) in a humidified 37℃ incubator containing 5% CO_2_. hFOB1.19 cells were cultured in DMEM/F12 medium (GIBCO) supplemented with 0.3 mg/ml G418 and 10% fetal bovine serum in a humidified 34℃ incubator containing 5% CO_2_.

### Real-time quantitative reverse transcriptase PCR (qRT-PCR)

Total RNA was isolated by TRIzol reagent (Thermo Fisher Scientific, Carlsbad, CA, USA). Nuclear and cytoplasmic RNA were separated using a Cytoplasmic & Nuclear RNA Purification Kit (Norgen Biotek, Thorold, ON, Canada). cDNA was synthesized with PrimeScript RT Reagent Kit (TaKaRa, Dalian, China). qRT-PCR assays were performed with SYBR Green PCR Kit (TaKaRa) and specific primers presented in Additional file 1: Table S2. The 2^−ΔΔCT^ method was adopted to analyze the relative gene expression with GAPDH served as an internal control. The back-splicing junction of circFNDC3B of the PCR products was verified by Sanger sequencing.

### Overexpression plasmids, shRNAs and transfection

Lipofectamine 3000 (Invitrogen) was used to transfect overexpression plasmids and shRNAs into OS cells. Full circFNDC3B sequence was constructed into a pcDNA3.1 (+) CircRNA Mini vector (Addgene, MA, USA) to generate the circFNDC3B overexpression plasmids. RNA binding motif protein 47 (RBM47), Insulin-like growth-factor-2 mRNA binding protein (IGF2BP1) or FNDC3B cDNAs were constructed into a pcDNA3.1 (+) vector to generate the overexpression plasmids. ShRNAs for circFNDC3B were designed and synthesized by GenePharma (Shanghai, China) and the target sequences were listed in Additional file 1: Table S3. ShRNAs for RBM47, IGF2BP1 and FNDC3B were purchased from Santa Cruz Biotechnology (sc-89082-SH, sc-40695-SH, sc-78339-SH, Dallas, Texas, USA). Overexpression or knockdown efficiency was assessed by qRT-PCR assay.

### CCK8 assay

5 × 10^3^ OS cells were seeded in 96-well plates, and 10 μl of CCK8 solution (Beyotime Biotechnology, Shanghai, China) was added at each time point after transfection. The absorbance at 450 nM was detected by a microtiter plate reader after incubation.

### 5-Ethynyl-2′-deoxyuridine (EdU) assay

2 × 10^5^ OS cells were seeded in 6-well plates and transfected with overexpression plasmids or shRNAs for 48 h when the cells reached 80–90% confluence. EdU assays were conducted using a Cell-Light EdU Apollo567 In Vitro Kit (Ribobio, Guangzhou, China). Cells were incubated with 50 μM EdU buffer at 37 °C for 2 h and Apollo dyeing reaction solution at room temperature for 30 min, followed by staining the nuclei with 4,6-diamidino-2-phenylindole (DAPI). Images were acquired using a fluorescence microscope to assess DNA replication activity via EdU-positive rates of cells.

### Wound-healing assay

2 × 10^5^ OS cells were seeded in 6-well plates and 10 μL pipette tips were used to scrape a straight scratch in the single-cell layer when the cells reached 80–90% confluence. The cells were then cultured with serum-free RPM-1640 medium for 24 h. Images of the wounds were captured at 0 h and 24 h after injury at the same wound location. ImageJ software was applied to calculate the wound-healing rate to evaluate the relative cell migration ability.

### Transwell invasion assay

Transwell invasion assays were conducted using transwell chambers (Corning, NY, USA) pre-coated with diluted Matrigel (Corning). OS cells transfected for 48 h were harvested and re-suspended in 200 μl serum-free RPM-1640 medium. The cell suspension was added to the upper chamber and complete medium (supplemented with 10% FBS) was added to the lower chamber. After 24 h, cells invading through the membrane were fixed with methanol followed by staining with 0.1% crystal violet. Five random fields per chamber were selected to count the invaded cell numbers and calculate the average. This assay was repeated three times independently for further statistical analysis.

### In vivo xenograft assay

The study for the animals was approved by the Experimental Animal Welfare and Ethics Committee of Affiliated Jinhua Hospital, Zhejiang University School of Medicine (Approval No. AL-JHYY202344). Lentivirus was used to stably overexpress circFNDC3B and RBM47 and silence FNDC3B in MG63 cells, as well as to silence circFNDC3B in 143B cells. Four male BALB/c mice (four-week-old) were subcutaneously injected with OS cells (1 × 10^7^). Tumor volume was recorded every week by the 0.5 × length × width^2^ method before the animals sacrificed by cervical dislocation.

### RNA fluorescence in situ hybridization (RNA-FISH)

RNA-FISH assay was performed using Alexa Fluor 594-labeled oligonucleotide probes specific for circFNDC3B junction sequence and Fluorescent In Situ Hybridization kit (RiboBio) according to the instructions provided by the manufacturer. Briefly, OS cells were fixed with 4% paraformaldehyde and treated with 0.5% Triton X-100. Then, cells were hybridized with circFNDC3B probe at 37 °C overnight in a hybridization chamber. DAPI was chosen for labeling cell nuclei and the images were acquired with a fluorescence microscope.

### Immunofluorescence

Cells seeded on coverslips were fixed with 4% paraformaldehyde, treated with 0.5% Triton X-100, and incubated with antibodies against RBM47 (PA5-52282, Invitrogen) or IGF2BP1 (712138, Invitrogen) at 4 °C overnight. Then, cells were incubated with goat anti-rabbit IgG-FITC antibody, followed by staining the nuclei with DAPI, at room temperature in the dark. The images were acquired with a fluorescence microscope.

### RNA binding protein immunoprecipitation (RIP) assay

RIP assay was carried out with an EZMagna RIP kit (Millipore, Billerica, MA, USA). Briefly, OS cells were lysed with RIP buffer and the lysates were incubated with magnetic beads conjugated with antibodies against RBM47 (orb630577, biorbyt, Wuhan, China), IGF2BP1 (712138, Invitrogen) or Normal Rabbit IgG (#2729, Cell Signaling Technology, Danvers, MA, USA) at 4 °C overnight. After the sample was digested with proteinase K, the immunoprecipitated RNA was isolated and analyzed by qRT-PCR assay.

### RNA stability analysis

To analyze the stability of RNA, first, RNA transcription of OS cells was blocked by treating with 10 μg/mL actinomycin D (Sigma-Aldrich, St. Louis, USA). Then, qRT-PCR assay was conducted to detect the leftover circFNDC3B and FNDC3B mRNA at different time points.

### Statistical analysis

All data from at least three independent experiments were presented as mean ± standard deviation (SD) and analyzed by the SPSS software (version 18.0, IBM, Chicago, IL, USA) or GraphPad Prism 5.0 software (GraphPad Software, La Jolla, CA, USA). Student’s t-test or one-way ANOVA analysis was performed to compare the difference between groups. *P* value < 0.05 was regarded as statistically significant.

## Results

### CircFNDC3B and FNDC3B mRNA were dysregulated in OS

**Fig. 1:**
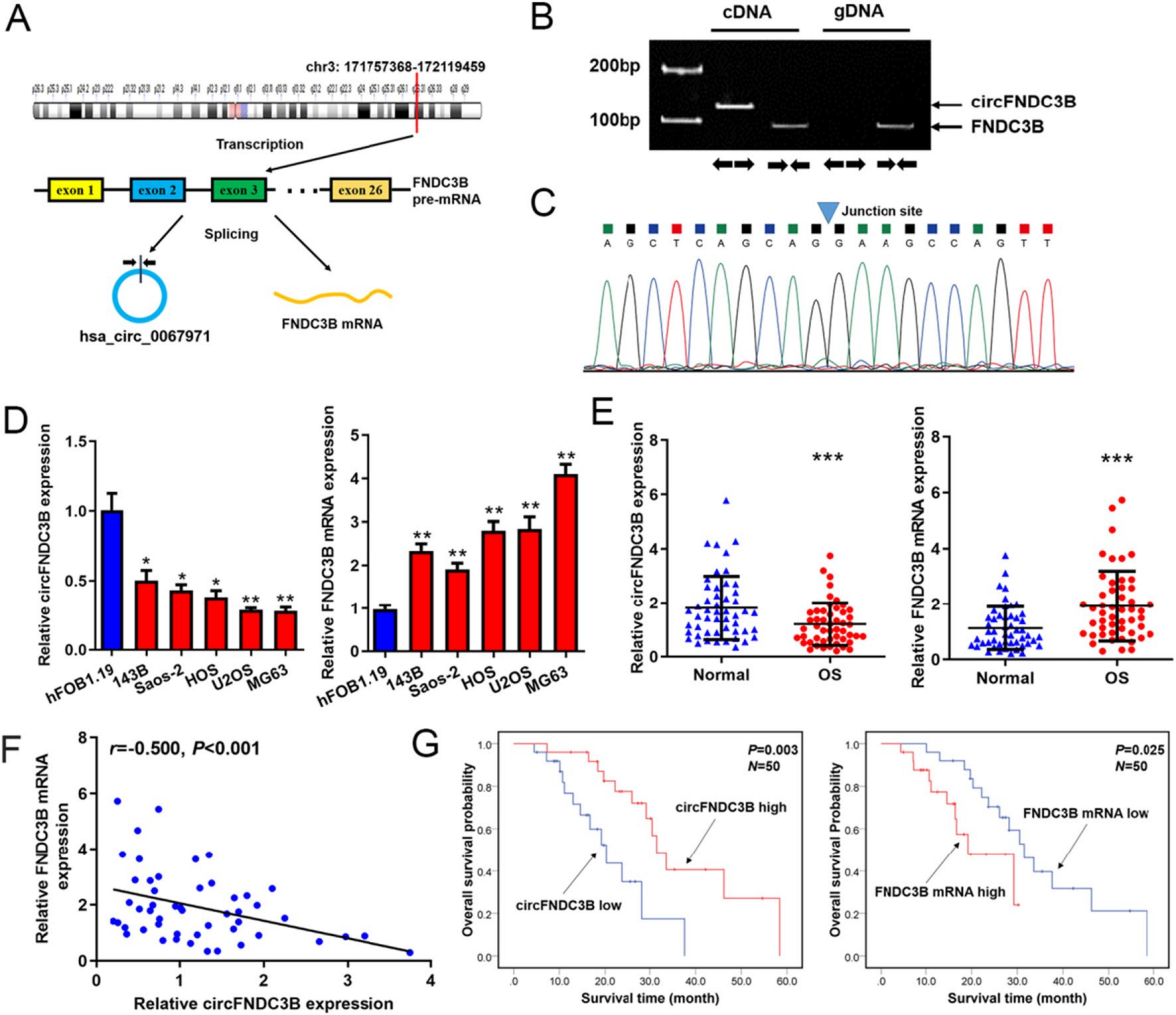
CircFNDC3B and FNDC3B mRNA were dysregulated in OS. **A** Schematic diagram exhibiting the origination of FNDC3B mRNA and circFNDC3B.** B** Divergent and convergent primers amplifying circFNDC3B and FNDC3B mRNA in cDNA and gDNA.** C** The back-splicing junction site of circFNDC3B was validated by sanger sequencing. **D, E** CircFNDC3B and FNDC3B mRNA levels in OS cell lines and tissues were detected by qRT-PCR. **F** Correlation between circFNDC3B and FNDC3B mRNA levels in OS tissues was analyzed by Pearson method. (**G**) Overall survival of OS patients analyzed with the Kaplan-Meier method and log-rank test. Data were shown as the mean ± SD, *P < 0.05, **P < 0.01, ***P < 0.001

The FNDC3B genomic locus was located in chr3: 171,757,368–172,119,459, and could be transcribed into a FNDC3B mRNA containing 26 exons (exon1-exon26) that may translate into a protein with 1204 aa (Fig. 1A). CircFNDC3B originated from the back-splicing of exon 2, which was located in chr3:171,830,241–171830380 region (Fig. 1A). Then, we designed divergent primers to detect circFNDC3B and convergent primers to detect the FNDC3B mRNA by qRT-PCR (Fig. 1B) and verified the back-splicing junction site of circFNDC3B using Sanger sequencing (Fig. 1C). Consistent with the profile of the previous study [13], our data also displayed that circFNDC3B expression was significantly decreased in OS cell lines compared with hFOB1.19 cell line (Fig. 1D). On the contrary, we found that FNDC3B mRNA was significantly upregulated in OS cell lines (Fig. 1D). In addition, we showed that the expression of circFNDC3B decreased while FNDC3B mRNA increased in OS tumor tissues (Fig. 1E) and the expression level of circFNDC3B and FNDC3B mRNA in OS tissues were negatively correlated with each (Fig. 1F). Abnormal circFNDC3B and FNDC3B mRNA expression were not only correlated with tumor size, Enneking stage and/or lymph node metastasis status (Table 1), but also associated with the prognosis of OS patients (Fig. 1G). These findings indicated that circFNDC3B and FNDC3B mRNA were dysregulated in OS and might be biomarkers for indicating disease progression and prognosis of OS patients.

**Table 1:** Correlation between the clinical variables of OS patients with circFNDC3B or FNDC3B mRNA expression

Clinical variables	*N*	circFNDC3B		*P*	FNDC3B mRNA		*P*
		Low	High		Low	High	
Gender							
Male	28	13	15		15	13	
Female	22	12	10	0.388	10	12	0.388
Age							
< 25y	33	14	19		17	16	
≥ 25y	17	11	6	0.116	8	9	0.500
Tumor size							
< 6 cm	29	10	19		22	7	
≥ 6 cm	21	15	6	0.010	3	18	0.000
Lymph node metastasis							
No	34	13	21		20	14	
Yes	16	12	4	0.016	5	11	0.064
Enneking stage							
I + IIA	35	14	21		20	15	
IB + III	15	11	4	0.031	5	10	0.108
Differentiation							
Well/moderately	31	15	16		16	15	
Poorly/undifferentiated	19	10	9	0.500	9	10	0.500
Location							
Femur/tibia	34	17	17		16	18	
Elsewhere	16	8	8	0.619	9	7	0.381

Data were analyzed by chi-square test

### CircFNDC3B suppressed OS progression in vitro and in vivo

**Fig. 2:**
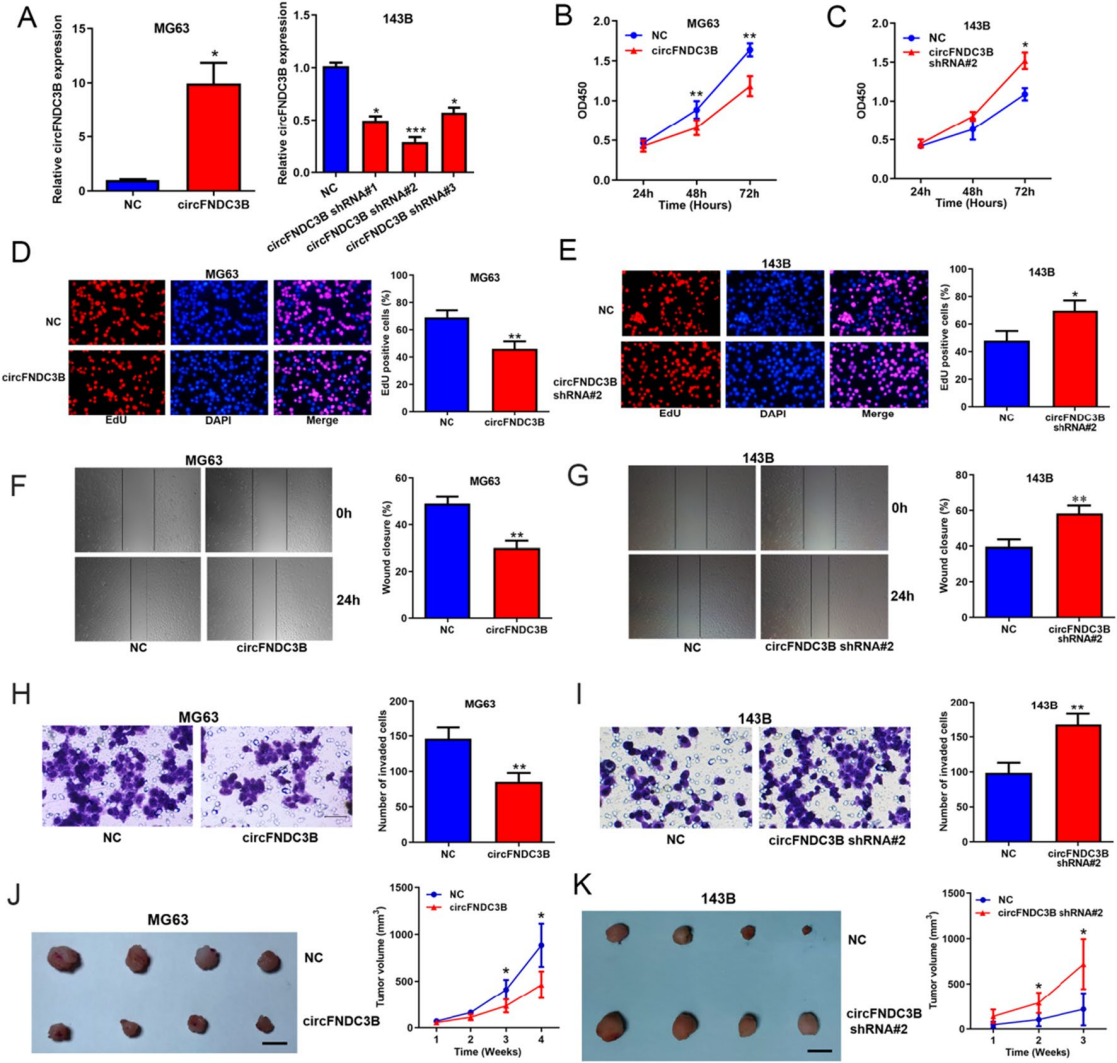
CircFNDC3B suppressed OS progression in vitro and in vivo. **A** Overexpression and knockdown efficiency of circFNDC3B in OS cells evaluated by qRT-PCR. CCK8 assays (**B, C**), EdU assays (**D, E**), wound-healing assays (**F, G**) and transwell assays (**H, I**) assessing the effects of circFNDC3B overexpression and knockdown on OS cells proliferation, migration and invasion. **J, K** Xenograft assays assessing the effects of circFNDC3B overexpression or knockdown on OS tumor growth in vivo; scale bar represents 1 cm. Data were shown as the mean ± SD, *P < 0.05, **P < 0.01, ***P < 0.001

Subsequently, we explored the biological functions of circFNDC3B in OS. MG63 cell line that presented the lowest circFNDC3B expression was selected to be transfected with overexpression plasmids and 143B cell line which presented the highest circFNDC3B expression was selected to be transfected with shRNAs (the shRNA#2 showed the highest silence efficiency) (Fig. 2A). Then, CCK8 and EdU assays displayed that overexpressing circFNDC3B restrained the proliferation ability of MG63 cells and silencing circFNDC3B promoted the proliferation capacity of 143B cells (Fig. 2B–E). Wound-healing and transwell assays showed that overexpressing circFNDC3B suppressed the migration and invasion ability of MG63 cells and silencing circFNDC3B accelerated the migration and invasion of 143B cells (Fig. 2F–I). In addition, the xenograft assays showed that overexpressing circFNDC3B significantly impeded tumor growth, and silencing circFNDC3B promoted tumor growth, in vivo (Fig. 2J and 2K). These data revealed that circFNDC3B suppressed OS progression both in vitro and in vivo.

### FNDC3B promoted OS progression in vitro and in vivo

**Fig. 3:**
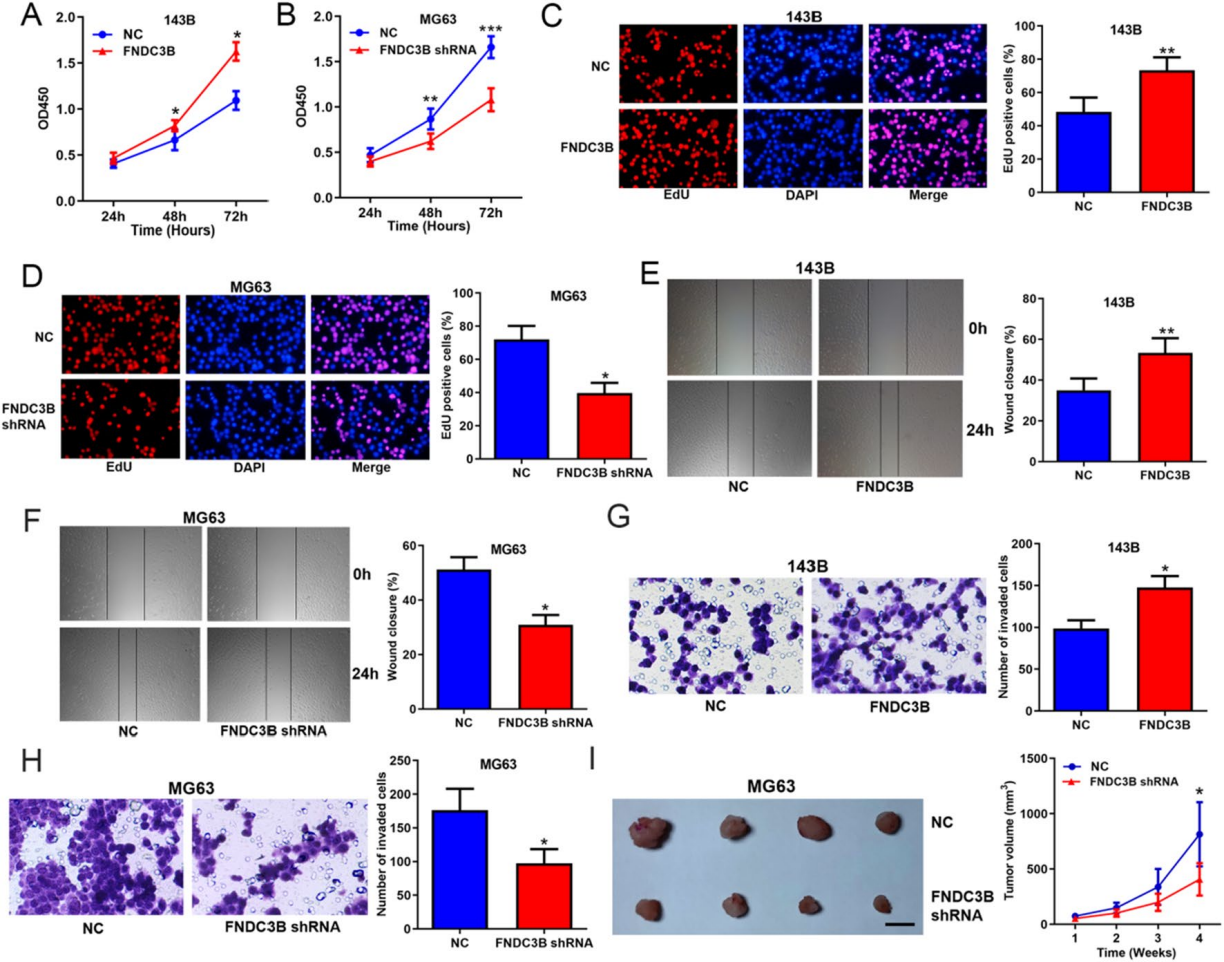
FNDC3B promoted OS progression in vitro and in vivo. CCK8 assays (**A, B**), EdU assays (**C, D**), wound-healing assays (**E, F**) and transwell assays (**G, H**) assessing the effects of FNDC3B overexpression and knockdown on OS cells proliferation, migration and invasion. **I** Xenograft assays assessing the effects of FNDC3B knockdown on OS tumor growth in vivo; scale bar represents 1 cm. Data were shown as the mean ± SD, *P < 0.05, **P < 0.01, ***P < 0.001

Accordingly, the results of CCK8 assays, EdU assays, wound-healing and transwell assays showed that overexpressing FNDC3B promoted while silencing FNDC3B suppressed the proliferation, migration and invasion of OS cells (Additional file 1: Figure S1, Fig. 3A–H). The xenograft assays demonstrated that knockdown of FNDC3B significantly restrained the tumor growth in vivo (Fig. 3I). These data revealed that FNDC3B played an opposite role with circFNDC3B in OS to promote malignant processes in vitro and in vivo.

### The circFNDC3B-FNDC3B mRNA imbalance was a consequence of alternative splicing of RBM47

**Fig. 4:**
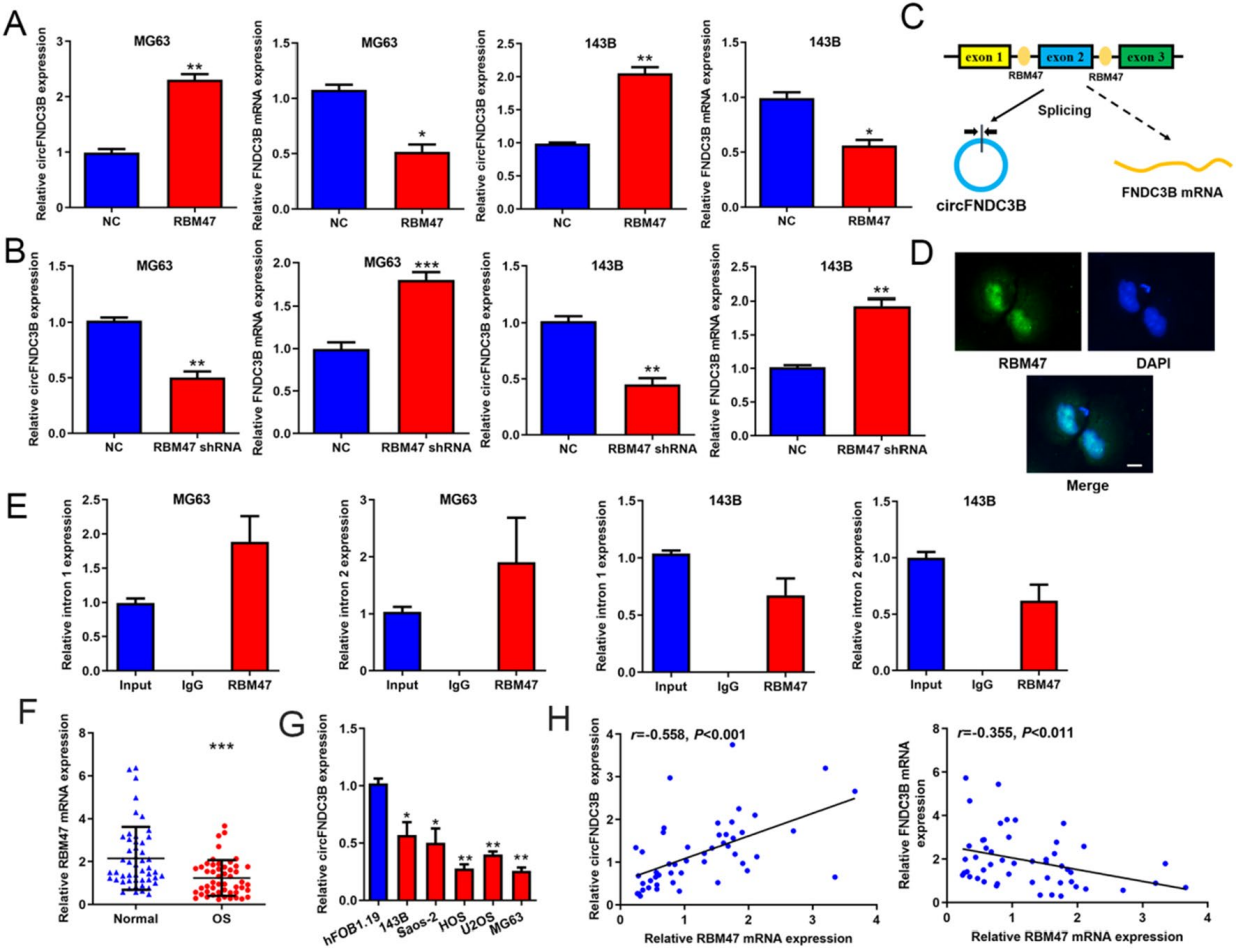
The circFNDC3B-FNDC3B mRNA imbalance was a consequence of alternative splicing of RBM47. **A, B** Regulation of RBM47 on circFNDC3B and FNDC3B mRNA expression evaluated by qRT-PCR. **C** Predicted regulation pattern of RBM47 on circFNDC3B-FNDC3B mRNA imbalance. **D** Subcellular localization of RBM47 protein displayed by immunofluorescence; scale bar represents 5 μm. **E** Interaction of RBM47 with intron 1 and intron 2 sequences validated by RIP assays. **F, G** RBM47 mRNA levels in OS cell lines and tissues were detected by qRT-PCR. **H** Correlation of RBM47 expression with circFNDC3B or FNDC3B mRNA levels in OS tissues were analyzed by Pearson method. Data were shown as the mean ± SD, *P < 0.05, **P < 0.01, ***P < 0.001

As we described above, the RBPs bind to the pre-mRNA introns and mediate alternative splicing may influence the generation of circRNAs. Here, we noticed that RBM47 was able to bind to the flanking introns (intron 1 and intron 2) of circFNDC3B using StarBase database (http://starbase.sysu.edu.cn) (Additional file 1: Figure S2). We also found that overexpressing RBM47 obviously raised the expression of circFNDC3B while decreased the FNDC3B mRNA level (Additional file 1: Figure S3, Fig. 4A); and knockdown of RBM47 inhibited the expression of circFNDC3B but increased the FNDC3B mRNA level (Additional file 1: Figure S3, Fig. 4B). Therefore, we raised a hypothesis that RBM47 bound to the flanking introns of circFNDC3B to facilitate the splicing of circFNDC3B, resulting in the reduction of FNDC3B mRNA (Fig. 4C). First, we observed that RBM47 was concentrated in nuclei of OS cells (Fig. 4D), which was the essential basis for playing the RNA splicing role. Then, OS cells were transfected with intron 1 and intron 2 expression plasmids and RBM47 antibody was used to conduct RIP assays. The results showed that both intron1 and intron2 were significantly enriched in the RBM47 immunoprecipitates (Fig. 4E), which confirmed the interaction of RBM47 with intron1 and intron2. In addition, we demonstrated that RBM47 mRNA expression was decreased in OS tissues and cell lines (Fig. 4F and G), and the RBM47 mRNA level in OS tissues was obviously correlated with both circFNDC3B and FNDC3B mRNA levels (Fig. 4H). Moreover, the correlation coefficient of RBM47 with circFNDC3B was larger than that of RBM47 with FNDC3B mRNA (Fig. 4H), which indicated that RBM47 mainly regulated the expression of circFNDC3B. Taken together, the above data suggested that RBM47 bound to the flanking introns of circFNDC3B to facilitate the splicing of circFNDC3B, resulting in the reduction of FNDC3B mRNA and the circFNDC3B-FNDC3B mRNA imbalance.

### RBM47 suppressed OS progression in vitro and in vivo

**Fig. 5:**
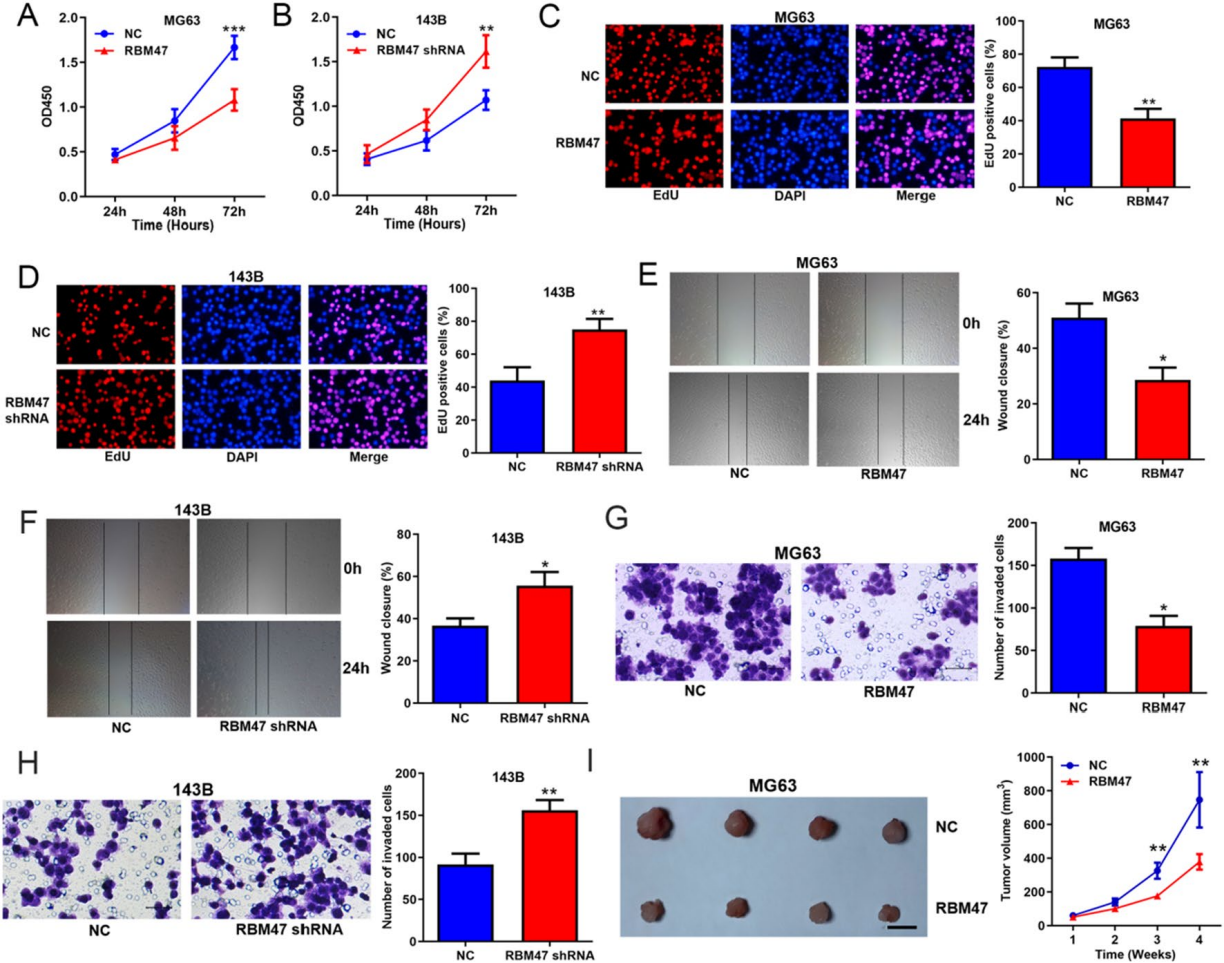
RBM47 suppressed OS progression in vitro and in vivo. CCK8 assays (**A, B**), EdU assays (**C, D**), wound-healing assays (**E, F**) and transwell assays (**G, H**) assessing the effects of RBM47 overexpression and knockdown on OS cells proliferation, migration and invasion. **I** Xenograft assays assessing the effects of RBM47 overexpression on OS tumor growth in vivo; scale bar represents 1 cm. Data were shown as the mean ± SD, *P < 0.05, **P < 0.01, ***P < 0.001

Since RBM47 was able to upregulate a tumor suppressor gene (circFNDC3B) and downregulate an oncogene (FNDC3B) in OS cells, we considered that it might exert anticarcinogenic effects in OS. Consistent with our conjecture, the in vitro and in vivo data exhibited that RBM47 suppressed OS progression (Fig. 5A–I).

### CircFNDC3B also reduced FNDC3B mRNA stability by competitively binding to IGF2BP1

**Fig. 6:**
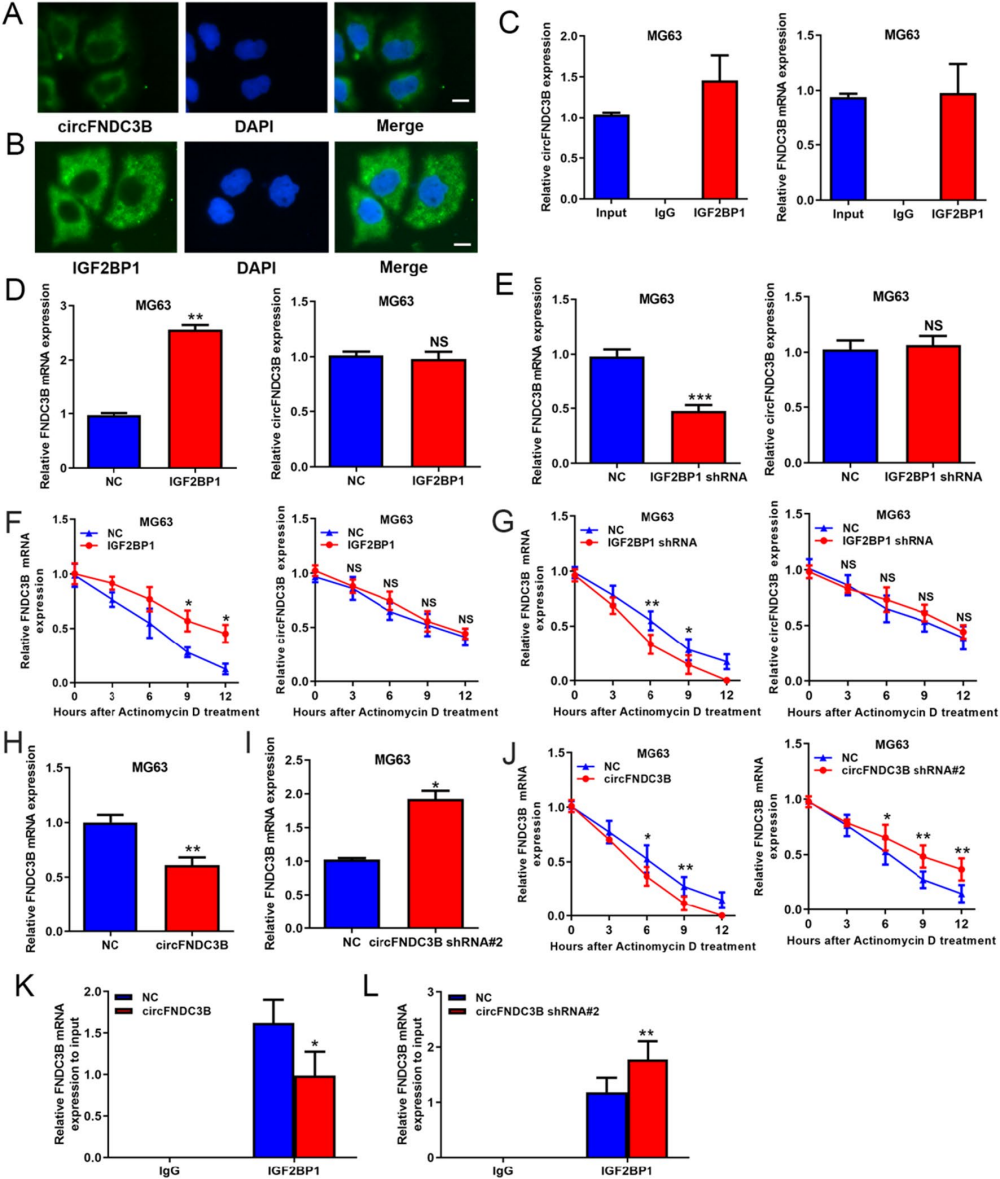
CircFNDC3B also reduced FNDC3B mRNA stability by competitively binding to IGF2BP1. **A** Subcellular localization of circFNDC3B displayed by RNA FISH; scale bar represents 5 μm. **B** Subcellular localization of IGF2BP1 protein displayed by immunofluorescence; scale bar represents 5 μm. **C** Interaction of IGF2BP1 with circFNDC3B and FNDC3B mRNA validated by RIP assays. **D, E** Regulation of IGF2BP1 on circFNDC3B and FNDC3B mRNA expression evaluated by qRT-PCR. **F, G** Influence of IGF2BP1 on circFNDC3B and FNDC3B mRNA stability. **H, I** Regulation of circFNDC3B on FNDC3B mRNA expression evaluated by qRT-PCR. **J** Influence of circFNDC3B on FNDC3B mRNA stability. **K,L** The influence of circFNDC3B overexpression or silence on the interaction of IGF2BP1 with FNDC3B mRNA was validated by RIP assays. Data were shown as the mean ± SD, *P < 0.05, **P < 0.01, ***P < 0.001

We further clarified the molecular mechanisms of circFNDC3B in regulating OS progression. We identified that circFNDC3B was concentrated in the cytoplasm of OS cells (Fig. 6A). Through the StarBase database, we notice that RBP IGF2BP1 was able to bind to both circFNDC3B and FNDC3B mRNA (Additional file 1: Figure S4), and we identified that IGF2BP1 was also concentrated in the cytoplasm of OS cells (Fig. 6B). Subsequently, RIP assays demonstrated that both circFNDC3B and FNDC3B mRNA were significantly enriched in the IGF2BP1 immunoprecipitates (Fig. 6C), which validated the interaction of IGF2BP1 with circFNDC3B and FNDC3B mRNA. Here, we found that overexpression or silence of IGF2BP1 (Additional file 1: Figure S5) significantly influenced the expression of FNDC3B mRNA but not circFNDC3B (Fig. 6D and E). Accordingly, RNA stability analysis also confirmed that IGF2BP1 significantly raised the stability of FNDC3B mRNA while showing no obvious influence on the stability of circFNDC3B (Fig. 6F and G). Furthermore, we revealed that overexpressing circFNDC3B decreased FNDC3B mRNA level and silencing circFNDC3B raised FNDC3B mRNA level (Fig. 6H, I). Accordingly, circFNDC3B significantly affected the stability of FNDC3B mRNA (Fig. 6J). We also presented that overexpressing circFNDC3B weakened the interaction between FNDC3B and IGF2BP1, and silencing circFNDC3B enhanced the interaction between FNDC3B and IGF2BP1 (Fig. 6K and L). To sum up, we concluded that both circFNDC3B and FNDC3B mRNA bound to IGF2BP1, and thereby, circFNDC3B reduced FNDC3B mRNA stability by competitively binding to IGF2BP1.

## Discussion

In this study, we identified a circFNDC3B/FNDC3B mRNA imbalance was involved in the malignant processes of osteosarcoma. CircFNDC3B was identified to be downregulated in OS cell lines compared with human osteoblast hFOB1.19 cell line by analyzing GEO datasets. Then, we verified the reduction of circFNDC3B in OS cell lines and tissues, and further discovered that circFNDC3B acted as a tumor suppressor gene to restrain OS progression in vitro and in vivo. On the contrary, FNDC3B mRNA was upregulated in OS cell lines and tissues, and acted as an oncogene to facilitate OS progression. FNDC3B has already been reported to promote tumor progression in various cancers via multiple mechanisms [17–22]. For example, FNDC3B was able to activate many oncogenic signaling pathways such as PI3K/mTOR signaling, Wnt/β-catenin signaling, Rb1 signaling and TGF-β signaling [18, 19, 22]. Here, we for the first time confirmed the oncogenic role of FNDC3B in OS.

Then, we clarified the mechanism aspect that account for the circFNDC3B/FNDC3B mRNA imbalance. We confirmed that RBM47 bound to the flanking introns of circFNDC3B to facilitate the splicing of circFNDC3B, resulting in the reduction of FNDC3B mRNA. RBM47 contains three classical RNA recognition motifs and was able to regulate pre-mRNA splicing and influence mRNA stability [23–25]. It has been reported to be downregulated in some cancers and suppress cancer progression [23, 25–28]. Here, in OS, we for the first time demonstrated that RBM47 was downregulated and acted as a tumor suppressor gene to restrain cancer progression in vitro and in vivo. We considered that RBM47 might exert anticarcinogenic effects by modulating circFNDC3B and FNDC3B mRNA.

In addition, our data demonstrated that both circFNDC3B and FNDC3B mRNA bound to IGF2BP1, and thereby, circFNDC3B reduced FNDC3B mRNA stability by competitively binding to IGF2BP1. IGF2BP1 is a RBP that modulates the metabolism of a variety of transcripts by enhancing RNA stability [29, 30]. In a large member of cancers, IGF2BP1 has been demonstrated to be highly expressed and facilitate the processes of cancers by modulating RNA molecules associated with cancer development [31, 32]. IGF2BP1 was uncovered to be upregulated in OS and promote malignant process by modulating the stability of some mRNAs [33, 34]. In this study, we showed that IGF2BP1 was able to enhance the stability of FNDC3B mRNA and increase the FNDC3B mRNA expression, whereas it exhibited no significant influence on circFNDC3B stability and expression. In other words, IGF2BP1 only promoted the expression of oncogene FNDC3B but not the expression of tumor suppressor gene circFNDC3B. However, the binding of circFNDC3B to IGF2BP1 may impede the interaction of IGF2BP1 with FNDC3B mRNA, and thereby FNDC3B mRNA stability. Because FNDC3B functions as an oncogene in many cancers and we also confirmed its malignant role in OS, we think circFNDC3B may exert tumor inhibitory effects by repressing FNDC3B mRNA.

## Conclusions

**Fig. 7:**
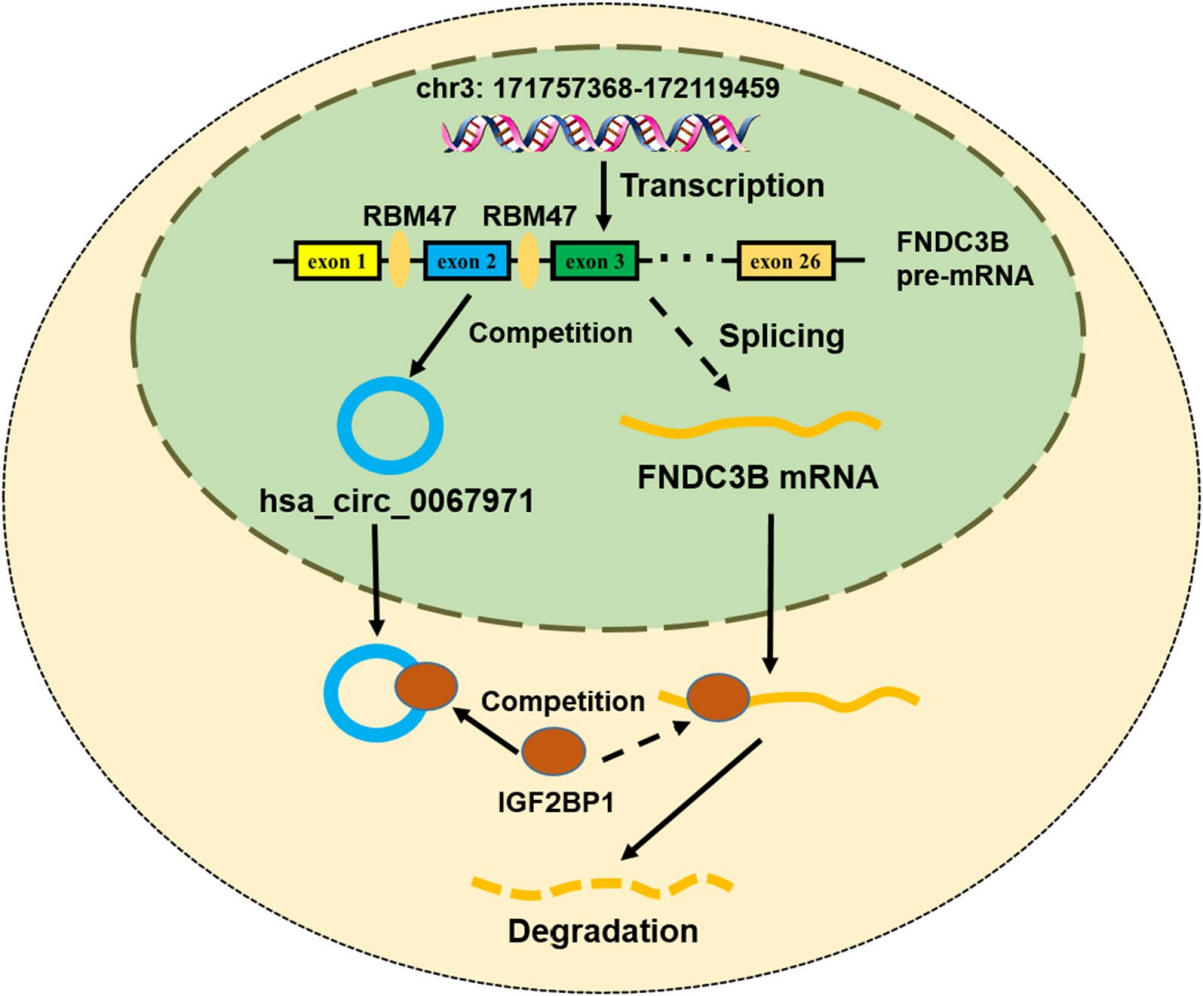
The schematic diagram exhibiting the RBM47 and IGF2BP1 mediated circular FNDC3B-FNDC3B mRNA imbalance in the malignant processes of OS

To summarize, our study found that circFNDC3B downregulated and FNDC3B mRNA upregulated in OS, which was a consequence of alternative splicing of RBM47. CircFNDC3B also inhibited FNDC3B mRNA expression by reducing its stability by competitively binding to IGF2BP1. The RBM47 and IGF2BP1 mediated circular FNDC3B/FNDC3B mRNA imbalance was involved in the malignant processes of osteosarcoma in vitro and in vivo (Fig. 7). This signaling might provide promising targets for therapeutic application of OS.

### Supplementary Information

**Additional file 1:** **Table S1**: Clinical characteristics of OS patients. **Table S2**: Primer sequences used in qRT-PCR. **Table S3**. Target sites of circFNDC3B shRNAs. **Figure S1**: Theconstruction of FNDC3B overexpressed or knockdown in OS cells. (A) Overexpression efficiency of FNDC3B in 143B OS cells assessed by qRT-PCR assay. (B) knockdown efficiency of FNDC3B in MG63 OS cells assessed by qRT-PCR assay. **Figure S2**: The binding sites of RBM47 on introns of circFNDC3B. (A) StarBase database was used to predict the binding sites of RBM47 on introns of circFNDC3B. **Figure S3**: The construction of RBM47 overexpressed or knockdown in OS cells. (A,B) Overexpression or knockdown efficiency of RBM47 in MG63 OS cells assessed by qRT-PCR assay. (C,D) Overexpression or knockdown efficiency ofRBM47 in 143B OS cells assessed by qRT-PCR assay. **Figure S4**: The Competitive binding sites of IGF2BP1 on circFNDC3B and FNDC3B mRNA. (A) StarBase database was used to predict the competitive binding sites of IGF2BP1 on circFNDC3B and FNDC3B mRNA. **Figure S5**: The construction of IGF2BP1 overexpressed or knockdown in OS cells. (A) Overexpression efficiency of IGF2BP1in MG63 OS cells assessed by qRT-PCR assay. (B) knockdown efficiency of IGF2BP1 in MG63 OS cells assessed by qRT-PCR assay.

## Data Availability

The datasets used in the current study are available from the corresponding author upon request.

## Abbreviations

CircRNAs: Circular RNAs
OS: Osteosarcoma
FNDC3B: Fibronectin type III domain containing 3B
RBM47: RNA binding protein RNA binding motif protein 47
IGF2BP1: Insulin-like growth-factor-2 mRNA binding protein
RBPs: RNA-binding proteins
QKI: Quaking
qRT-PCR: Real-time quantitative reverse transcriptase PCR
EdU: 5-Ethynyl-2′-deoxyuridine
RNA-FISH: RNA fluorescence in situ hybridization
RIP: RNA binding protein immunoprecipitation
