# Supplementary material for: A RBM47 and IGF2BP1 mediated circular FNDC3B-FNDC3B mRNA imbalance is involved in the malignant processes of osteosarcoma
Source: Cancer Cell Int. 2023 Dec 21;23:334. doi: 10.1186/s12935-023-03175-3 (PMC10740216; doi:10.1186/s12935-023-03175-3)
Supplement: Supplementary file 1 — Additional file 1: Table S1: Clinical characteristics of OS patients. Table S2: Primer sequences used in qRT-PCR. Table S3. Target sites of circFNDC3B shRNAs. Figure S1: Theconstruction of FNDC3B overexpressed or knockdown in OS cells. (A) Overexpression efficiency of FNDC3B in 143B OS cells assessed by qRT-PCR assay. (B) knockdown efficiency of FNDC3B in MG63 OS cells assessed by qRT-PCR assay. Figure S2: The binding sites of RBM47 on introns of circFNDC3B. (A) StarBase database was used to predict the binding sites of RBM47 on introns of circFNDC3B. Figure S3: The construction of RBM47 overexpressed or knockdown in OS cells. (A,B) Overexpression or knockdown efficiency of RBM47 in MG63 OS cells assessed by qRT-PCR assay. (C,D) Overexpression or knockdown efficiency ofRBM47 in 143B OS cells assessed by qRT-PCR assay. Figure S4: The Competitive binding sites of IGF2BP1 on circFNDC3B and FNDC3B mRNA. (A) StarBase database was used to predict the competitive binding sites of IGF2BP1 on circFNDC3B and FNDC3B mRNA. Figure S5: The construction of IGF2BP1 overexpressed or knockdown in OS cells. (A) Overexpression efficiency of IGF2BP1in MG63 OS cells assessed by qRT-PCR assay. (B) knockdown efficiency of IGF2BP1 in MG63 OS cells assessed by qRT-PCR assay. [file 12935_2023_3175_MOESM1_ESM.docx]

Table S1. Clinical characteristics of OS patients

| Characteristics | Range/*N* (%) |
| --- | --- |
| Gender (Male/Female) | 28 (56%)/22 (44%) |
| Age (<25y/≥25y) | 33 (66%)/17 (34%) |
| Height (cm) | 115-201 |
| Body weight (kg) | 27-96 |
| Smoking (No/Yes) | 31 (62%)/19 (38%) |
| Tumor size (<6cm/≥6cm) | 29 (58%)/21 (42%) |
| Lymph node metastasis (No/Yes) | 34 (68%)/16 (32%) |
| Enneking stage (I+IIA/IB+III) | 35 (70%)/15 (30%) |
| Differentiation (Well+moderately/ Poorly+undifferentiated) | 31 (62%)/19 (38%) |
| Location (Femur+tibia/Elsewhere) | 34 (68%)/16 (32%) |
| Pathologically fracture (No/Yes) | 9 (18%)/41 (82%) |
| Pathology (Osteoblastic/Chondroblast/Fibroblastic/ Telangiectatic) | 20 (40%)/17 (34%)/11 (22%)/2 (4%) |

Table S2. Primer sequences used in qRT-PCR

| Genes | Forward primer | Reverse primer |
| --- | --- | --- |
| circFNDC3B | TTGCTGAACGGAGAGGTAGC | GAGGGATTTGGTCGGTCATCA |
| FNDC3B | ACAATGGCTATGGGAAGGGC | TGCTCGTCGCTCTGTTTTCT |
| RBM47 | CAAGGAAGTGAGGTGTGCAA | ACACAGTCAGGGGATGAGGA |
| GAPDH | CAATGACCCCTTCATTGACC | TTGATTTTGGAGGGATCTCG |

Table S3. Target sites of circFNDC3B shRNAs

| shRNAs | Target sequence (5’-3’) |
| --- | --- |
| shRNA#1 | ATGCAGCTCAGCAGGAAGCCA |
| shRNA#2 | GCTCAGCAGGAAGCCAGTTGA |
| shRNA#3 | CAGCTCAGCAGGAAGCCAGTT |

**Figure S1. The construction of FNDC3B overexpressed or knockdown in OS cells.**


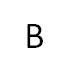

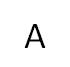

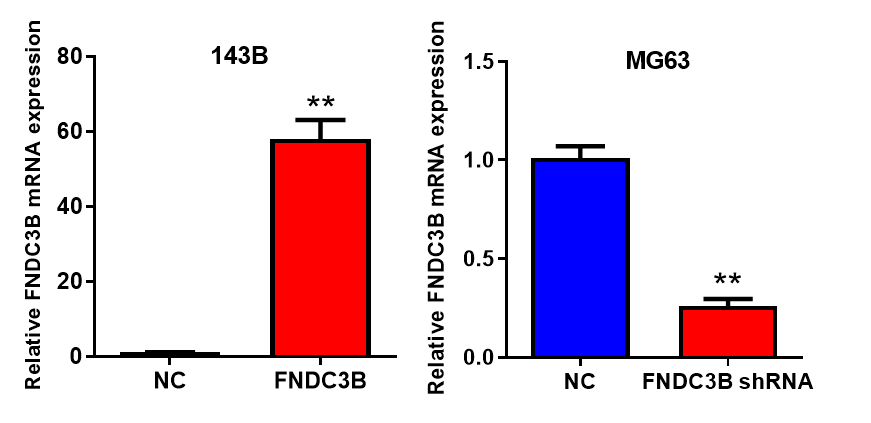


Figure S1. (A) Overexpression efficiency of FNDC3B in 143B OS cells assessed by qRT-PCR assay. (B) knockdown efficiency of FNDC3B in MG63 OS cells assessed by qRT-PCR assay.

**Figure S2. The binding sites of RBM47 on introns of circFNDC3B.**


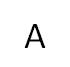

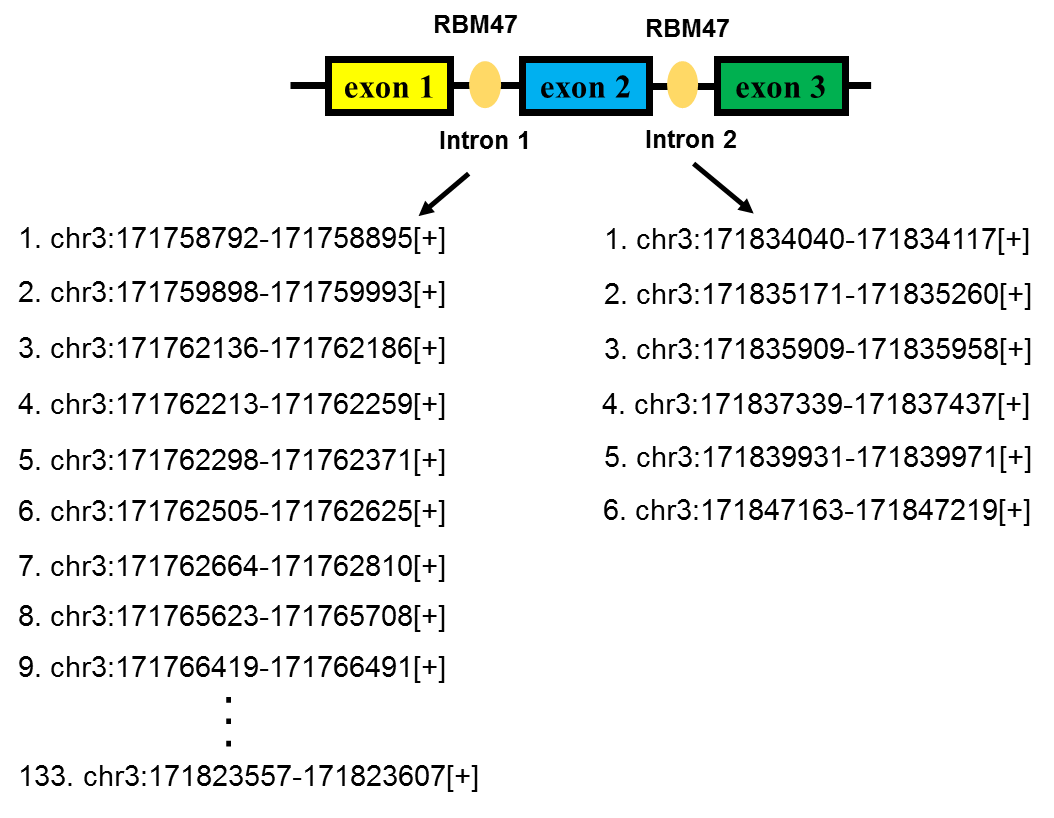


Figure S2. (A) StarBase database was used to predict the binding sites of RBM47 on introns of circFNDC3B.

**Figure S3. The construction of RBM47 overexpressed or knockdown in OS cells.**


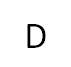

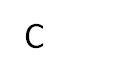

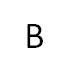

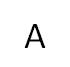


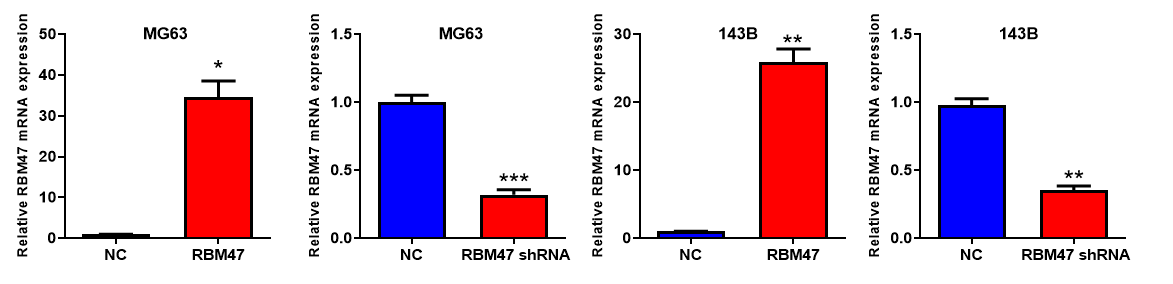


Figure S3. (A,B) Overexpression or knockdown efficiency of RBM47 in MG63 OS cells assessed by qRT-PCR assay. (C,D) Overexpression or knockdown efficiency of RBM47 in 143B OS cells assessed by qRT-PCR assay.

**Figure S4. The Competitive binding sites of IGF2BP1 on circFNDC3B and FNDC3B mRNA.**


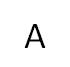


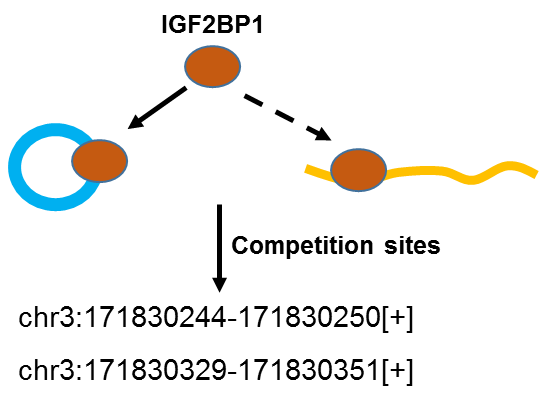


Figure S4. (A) StarBase database was used to predict the competitive binding sites of IGF2BP1 on circFNDC3B and FNDC3B mRNA

**Figure S5. The construction of IGF2BP1 overexpressed or knockdown in OS cells.**


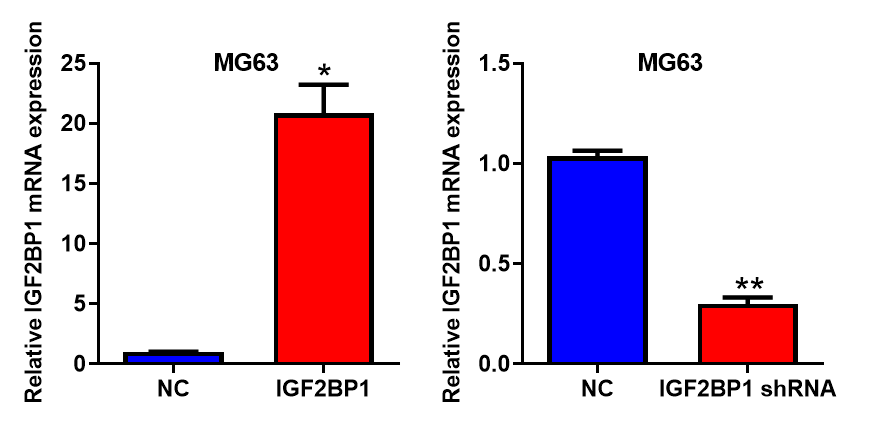


Figure S5. (A) Overexpression efficiency of IGF2BP1in MG63 OS cells assessed by qRT-PCR assay. (B) knockdown efficiency of IGF2BP1 in MG63 OS cells assessed by qRT-PCR assay.
